# Secretive and close? How sharing secrets may impact perceptions of distance

**DOI:** 10.1371/journal.pone.0233953

**Published:** 2020-06-11

**Authors:** Mariela E. Jaffé, Maria Douneva

**Affiliations:** Center for Social Psychology, University of Basel, Basel, Switzerland; Mälardalen University, SWEDEN

## Abstract

Having secrets is incredibly common. However, secrecy has only recently started to receive more attention in research. What has largely been neglected so far are the consequences of secret-sharing for the relationship between sharer and receiver. In this project, we aim to fill this gap. Previous research has shown that closeness can make secret-sharing more likely. Building on research from the self-disclosure and relationship literature, we experimentally investigate whether secret-sharing might in turn increase perceptions of closeness. In addition, we test the valence of the secrets shared as potential driver of the hypothesized effect, as negative secrets might signal a high level of trust, but might also present a burden to the receiver. To provide a holistic picture, we build on a variety of methods to investigate three perspectives: Study 1 focuses on the receiver and tests whether another person sharing secrets (vs. nonconfidential information) decreases distance in the eyes of the receiver. Study 2 investigates lay theories and tests how an observer perceives the relationship between two people who share secrets (vs. nonconfidential information). Study 3 tests whether these lay theories about sharing secrets are predictive of behavior, and how a sharer might choose secrets of negative or positive valence to decrease perceived distance on the receiver’s side. Our results will contribute to the understanding of how sharing secrets affects the way individuals think about each other, how close they feel to each other, and how they interact with each other.

## Introduction

We all have our personal secrets; stories from the past or present that we do not want to share with other people. In his famous novel *Ulysses*, James Joyce reflects on this fact and points out that “secrets, silent, stony sit in the dark palaces of both our hearts” [1]. Secrets have not only sparked the interest of writers, but also of scientists. Researchers have defined secrecy as “an intention to conceal information from one or more individuals” [2] and note that it is incredibly common. Slepian and colleagues [2] found that almost all of their study participants reported currently having at least one secret. These secrets often concerned extra-relational thoughts, sexual behavior, lies, and romantic desires not shared with anybody, but also abortion, sexual orientations, and marriage proposals, which are not kept entirely to oneself but are not openly shared either.

Turning back to Ulysses, James Joyce does not only observe that we all have secrets, but describes individuals as “weary of their tyranny” and secrets as “tyrants willing to be dethroned” [1]. His observation is in line with research showing that there is a tension between revealing and concealing private information [3]. Having secrets can result in negative consequences for the secret-keeper: Keeping a secret is associated with rumination due to suppression of thoughts and more intrusive thinking [4, 5], it can be stressful, and can harm health [6]. Furthermore, it is not necessarily only the concealment of secrets, but also the associated increase in experiencing fatigue [7] and more frequent spontaneous thoughts about the secrets that negatively impact individuals’ well-being [2, 8]. Researchers have also investigated the antecedents and consequences of “dethroning the weary tyrants” [1] by sharing a secret with another person. Deciding to share a secret is not as uncommon as one might think: Sharing a secret with at least one or two persons seems to be at least as common as not sharing a secret at all [2]. It appears that although there are reasons to keep a secret, there are also reasons for sharing it.

Previous research has provided some explanations for why individuals might want to share a secret. One line of research has investigated how disclosing a secret impacts the secret-sharer. Revealing personal secrets, even if only in writing, seems advantageous [6, 9]: Disclosing secrets may ease worry [10], decrease distress when experiencing intrusive thoughts [5], increase self-esteem [11] and well-being [12], and lower discomfort and tension [13]. Another line of research has investigated a different perspective, namely the impact of sharing a secret on the receiver. Receivers of secrets may experience increased intimacy, but at the same time a burden and negative feelings while guarding those secrets [14]. The consequences may depend on the size and severity of the secrets: The secret’s importance, its negative valence, and the negative face threat of secret-keeping (e.g., as being asked to keep a secret leads to behavioral constraints) are all positively associated with cognitive burden for the receiver [15]. A third line of research aims to integrate both perspectives, namely those of the secret-keeper and the secret-receiver, and points out that there are circumstances under which the secret-keeper is better off keeping the secret to him- or herself, for an overview see [16]. Critical to this could be the recipient’s reaction towards the disclosure of the secret: Afifi and Caughlin [11] showed that rumination among individuals who reveal a secret is only reduced when they perceive the receiver’s reaction as positive.

As outlined above, previous research has primarily focused on the impact of disclosing secrets on the secret-sharer or on the receiver individually. However, the disclosure of a secret involves at least two people and cannot exist in isolation. It therefore appears plausible that not only the individuals, but also the relationship between sharer and receiver should be affected by the exchange of the secret. Still, this aspect has often been neglected. In this article, we aim to fill this gap by focusing on the effects of sharing secret information about oneself on the perceived social distance between secret-sharers and receivers. To this end, we investigate potential changes in the relationship from three perspectives, implementing a variety of methods: First, we aim to understand the receiver’s perspective when being told secrets of positive and negative valence compared to nonconfidential information using audio recordings of phone calls as well as asking for participants’ experiences (Study 1). Next, we aim to understand how an observer would judge the effects of secret-sharing on the relationship between sharer and receiver using written vignettes (Study 2). Both studies allow us to investigate individuals’ lay theories, meaning individuals’ assumptions about “the nature of the self and the social world” [17]. Individuals might use lay theories to derive hypotheses to predict, but also to understand and interpret their social world in an efficient way [18]. Going beyond the interpretation of the social world, lay theories impact individuals’ perceptions and actions [18], more specifically in social relationships [19]. In the final study we therefore aim to understand how sharers might deliberatively choose to share (negative or positive) secrets in cases where they want to create social closeness or distance between themselves and another person (Study 3). This approach also allows to investigate whether the lay theories examined in the study on the observer’s perspective might transfer to individuals’ actual choices and behavior when sharing information. Predictions and expectations of actors and observers might be asymmetric [20], meaning that people who self-disclose fears, anxieties, or vulnerabilities might overestimate the recipient’s negative reaction [21, 22]. Observers might therefore have a different take on the impact of sharing secrets on the social distance between interaction partners than the secret-sharers themselves.

Next to looking at the effects of secret-sharing from these three perspectives, we also use a variety of methods to cross-validate our findings, as “each type of method, considered alone, is imperfect” [23] and implementing different methods increases confidence in and generalizability of our findings. To this end, we use audio recordings to assess individuals’ reactions in a setting with higher ecological validity, which we complement by asking for descriptions of personal experiences (Study 1). Furthermore, we include written vignettes, which increase the internal validity of our study due to the higher level of standardization. Last, we investigate goal-directed behavior to understand not only individuals’ perceptions but also their actions when sharing secrets. All in all, this work provides an integrative view on how secret-sharing affects the relationship between individuals.

### Social distance and sharing secrets

An action such as the disclosure of a secret presumably interplays with social distance between the sharer and the receiver. Social distance is a measure of intimacy between two people, meaning that it reflects how close or distant they feel to each other [24, 25]. While a person’s best friend is socially very close, a stranger is socially distant. Previous research has already identified social distance as an antecedent of revealing secrets, which will be discussed below. However, the consequences of revealing secrets on social distance between interaction partners are less clear and ask for further research, and are therefore the focus of our suggested empirical study plan.

So far, a few studies have established the link that psychological closeness may lead to the disclosure of secrets. Jourard and Friedman [26] found that participants disclose more information as an experimenter reduced the distance to the participants by self-disclosing personal information (which could also be interpreted as reducing social distance) as well as establishing minimal physical contact. These early findings are corroborated by more recent ones, showing that the closer social network ties individuals had, the more secrets were confided in them [27]. Apparently, individuals are more likely to reveal secrets to people to whom they have close social ties, meaning to people who are spatially and socially close to them. Furthermore, being compassionate and assertive predicts having secrets confined in oneself [27] and one could speculate that compassion could signal psychological proximity.

### Would the disclosure of secrets impact social distance?

As outlined above, confiding in psychologically close others seems more likely than revealing secrets to psychologically distant others or strangers. The link between closeness and confiding secrets, however, does not need to be unidirectional, meaning that revealing secrets may also impact perceptions of psychological closeness, see [28]. Previous research investigating self-disclosure indicates that sharing personal information—which can but is not necessarily secret—is important for developing closeness and gaining emotional support in relationships [29, 30]. This is also the core assumption of Social Penetration Theory [31], which provides a framework describing the development of interpersonal relationships, with the metaphor of an “onion model” [32]. More specifically, the theory proposes that layers of personal information are “peeled back” over time [32] by increasing disclosure [30] and that this self-disclosure increases intimacy to a certain point [32]. Furthermore, it has been found that sharing extraordinary compared to ordinary experiences enhances closeness, but that discomfort associated with the interaction is necessary for this effect to occur [33]. The act of sharing a secret usually involves both: sharing something extraordinary, and experienced discomfort while sharing.

Interestingly, self-disclosure can also serve as a form of boundary regulation, as individuals control how much (or how little) contact they maintain with others [34]. As described before, self-disclosure can be used to develop closeness in a relationship; see also research on the experimental generation of interpersonal closeness [35, 36]. By communicating in a warm way with distant others, distance can be bridged and perceived closeness increased [37]. On the other hand, if individuals decide to not share or to even actively hide information, they might create (physical) distance between themselves and other persons by avoiding to meet them [34].

However, it is not only self-disclosure in general, but more specifically the disclosure of one’s secrets that can signal the development of a closer relationship [38], which may result in perceptions of reduced social distance. Based on this research, we hypothesize that social distance between a sharer and receiver is not only a predictor, but also a consequence of sharing secrets.

### Importance of the research question

Many, if not all of us, carry one or more secrets. Some of them may prove to be rather insignificant if others would find out about them, whereas others might profoundly change the way others perceive us (e.g., when confessing infidelity towards one’s partner). Whether or not one should share a secret with someone is a question that research has often examined from the individual’s point of view (e.g., will the secret-sharer ruminate less after telling a friend?). However, as individuals usually share secrets with close others who they are likely to interact with in the future, it is critical to take into account how people expect their relationship with the other person to be influenced by the act of sharing a secret. Individuals’ beliefs and expectations regarding this change in the relationship most probably influence whether they themselves are willing to trust and confide in somebody else.

As our suggested studies focus on different perspectives, our results will not only contribute to the understanding of individuals’ lay theories with regards to secret-sharing or secret-receiving, but will also allow us to make predictions about the way individuals think about each other, how close they feel toward each other, and how they will eventually interact in the future after one has shared a secret with the other. At the same time, our research directly addresses gaps in the information-sharing literature that have recently been voiced, for example under which conditions sharing positive information with others leads to positive consequences [39], or whether people can accurately predict consequences of sharing information with others [39].

### Proposed hypotheses and research plan

Our overall hypothesis is that the revelation of secrets impacts perceptions of change in social distance between the secret-sharer and the receiver. More specifically, we predict that sharing secrets (compared to nonconfidential information) about oneself *decreases* perceived social distance between the two interaction partners (Hypothesis 1).

We will also investigate, in an exploratory manner, whether the valence of the information shared might make a difference for the receiver (Hypothesis 2). Previous research has shown that it is not the intention but the outcome of a behavior that impacts distance perceptions [40], which leads to the speculation that the hypothesized decrease in social distance depends on the nature of the secrets revealed (the “outcome”). Vrij, Paterson, Nunkoosing, Soukara, and Oosterwegel [41] cautioned against making strong claims about the benefits of disclosure without acknowledging that disclosing information can also be associated with negative aspects. Zhang and Dailey [15] showed that the valence of the shared secret is associated with relational distancing initiated by the receiver of the secret. Sharing positive personal information may result in feelings of closeness and therefore bridge psychological distance. However, sharing a “dark” secret about oneself might result in negative reactions on the side of the receiver and therefore, in turn, result in negative consequences for the sharer [11]. In consequence, the receiver and sharer could decide to increase social (or more general: psychological) distance between themselves to reduce the intensity of negative affect [42]. In contrast, sharing a “dark” secret my signal higher levels of trust and vulnerability to the receiver, and the disclosure could then result in higher levels of liking [43] and thereby enhance social closeness. We therefore test for differential effects of positive and negative secrets. As derived from previous literature and outlined above, sharing a negative secret could a) have the same effect as sharing a positive secret, namely to decrease social distance, b) have an even stronger effect than sharing a positive secret, namely to decrease social distance even more, or c) be associated with an *in*crease in perceived social distance between interaction partners. Each one of those outcomes would be of interest to better understand effects of valence when sharing secrets.

We aim to test our assumptions by taking three perspectives in three studies. Throughout the studies, we compare a secret condition to a nonconfidential condition. To ensure comparability between the conditions’ content and to rule out alternative explanations for our findings (such as that effects are driven by previous levels of closeness or different information shared), we use content-wise identical information in both conditions but either frame it as secret or as general news. In Study 1, participants will be at the receiver’s end of the sharing process, and will be asked to listen to an audio recording presented as a phone call in which one person (i.e., the secret-sharer) tells them a secret or news about him-/herself. The audio recording will contain a brief description of the situation and the content of the sharer’s revelation, and participants will subsequently be asked to evaluate their relationship with the secret-sharer. Furthermore, we will exploratorily tap into participants’ own experiences by asking them to recall a situation in which somebody shared a secret (vs. news) with them and whether this sharing was associated with a change in distance between them. In Study 2, participants will take the perspective of an external observer by reading a brief scenario in which two other people exchange information. After reading the scenario, they will then be asked to evaluate the perceived distance between these two people. As Study 2 focuses on individuals’ lay theories, we will then test whether lay theories might also impact participants’ choices and behaviors when sharing a secret themselves. In Study 3, participants will therefore be invited to the lab and put into the position of a secret-sharer, where we aim to understand what type of information they share when their objective is to decrease versus increase social distance.

### Initial support from an informal pretest

We conducted a pretest to gain preliminary insight and support for our main hypothesis that sharing secrets decreases perceived social distance. In this pretest, we asked participants to take the receiver’s position and read a vignette in which a woman shares a piece of information about a new job offer with them (translated Vignette 1 in S1 File, reframed as a story that Helen tells the participant). As we intend to do in all studies, we presented all participants with the same information, but we either clearly framed it as a secret in the secret condition or news in the nonconfidential condition. Participants were then asked to imagine the situation and reflect on the information by describing where the situation might have taken place and how they would react towards the information. Subsequently, all participants rated whether they would feel relaxed versus tense, well versus unwell, secure versus insecure, happy versus sad, and satisfied versus dissatisfied (semantic differential; 5-point scale) in the situation. Participants were then asked to evaluate the imagined social distance between themselves and the woman in the vignette on the Inclusion of Other in the Self Scale [44]. This scale served as a pictorial measure of closeness ranging from 1 = *interpersonally distant* to 7 = *interpersonally close*. We recruited 103 German-speaking participants via Clickworker for this pretest. Prior to data analysis, we excluded two participants who had asked for their data to be excluded or reported insufficient language proficiency. We excluded seven further participants when screening their descriptions of the scene as two answers was not readable or not in German and five other answers were identical and not informative of the scenario, giving us reason to believe that one person completed the survey several times. The dataset, analysis script, and original materials can be found online: https://osf.io/m68sr/.

The resulting sample consisted of 38 female and 56 male participants (*M*_*age*_ = 41.67; *SD*_*age*_ = 13.09). The descriptive results indicate that participants in the secret condition (*n* = 48) compared to participants in the news condition (*n* = 46) imagined feeling more relaxed (*M* = 2.69 vs. *M* = 2.91), well (*M* = 2.23 vs. *M* = 2.46), more secure (*M* = 2.29 vs. *M* = 2.50), happier (*M* = 2.31 vs. *M* = 2.43), and more satisfied (*M* = 2.04 vs. *M* = 2.26). Most importantly, participants in the secret condition imagined feeling closer to the person in the vignette compared to participants in the news condition (*M* = 4.85 vs. *M* = 4.30). For a better overview, all results are summarized in Table 1.

**Table 1:** Overview of results from pretest.

	Condition			
Dependent Variable	Secret *M* (*SD*)	News *M* (*SD*)	*p*-value (two-sided)	*d*
Reaction towards information shared				
… relaxed (1) versus tense (5)	2.69 (1.26)	2.91 (1.19)	.374	0.18
… well (1) versus unwell (5)	2.23 (1.10)	2.46 (1.17)	.333	0.20
… secure (1) versus insecure (5)	2.29 (1.17)	2.50 (1.28)	.411	0.17
… happy (1) versus sad (5)	2.31 (1.13)	2.43 (1.31)	.629	0.10
… satisfied (1) versus dissatisfied (5)	2.04 (0.82)	2.26 (0.86)	.209	0.26
Evaluation of distance				
… interpersonally distant (1) to close (7)	4.85 (1.53)	4.30 (1.58)	.089	-0.35

These descriptive differences do not reach statistical significance (presumably due to the lack of power to detect a small effect of *d* = -.35). We believe that for the main studies it is therefore important to consider the effect size by not only increasing the sample size but also by increasing the number of trials and asking participants to rate more than one situation.

The main purpose of the pretest was to provide us with critical insights regarding the direction of the effect, which is in line with our assumptions, and regarding the design of the subsequent studies. It is noteworthy that we observed a pattern in line with our assumptions despite the hypothetical nature of the study (i.e., participants neither recalled real instances nor did they interact with a real person, instead they only imagined a situation). All in all, the descriptive differences provide a promising outlook with regard to the three studies suggested in this article by lending initial support to our main hypothesis.

## Study 1

Study 1 serves to investigate whether a person sharing information about her- or himself that is either framed as a secret or not will impact participants’ perceptions of social distance between them and the person. To this end, participants in Study 1 will be asked to listen to audio recordings that are presented as phone calls. All audio recordings will include a person describing a situation in which they tell the participants a piece of information that is, in the end, framed as a secret or not. This information is furthermore either positive or negative. Participants are asked to carefully listen to these audio recordings and to subsequently judge potential changes in social distance between themselves and the person sharing the information with them. After evaluating all vignettes, we will ask them to recall a situation in which somebody shared a secret (news) with them and how this incident impacted social distance.

Study 1 will allow testing Hypotheses 1 and 2: Exchanging a secret reduces perceptions of social distance more than exchanging a piece of news (Hypothesis 1), but eventually to a different extent in the case of positive compared to negative secrets (Hypothesis 2). The setup allows testing our hypotheses in both an experimental setup with standardized material (audio recordings which are content-wise identical between conditions) and more ecologically valid material (idiosyncratic experiences but also materials that mimic interpersonal everyday exchanges).

### Methods

An approval by the University’s IRB (#20-18-1) has been obtained for the draft of all of the studies and we will report all measures, manipulations, and exclusions.

#### Participants

The study will be conducted as an online study advertised as a *study on the evaluation of behavior* via the online participant recruitment platform Prolific. Our a priori power analysis with an α-level of .05, a desired power of .95, and a small to medium effect size estimate (*f* = 0.15) while assuming a small correlation between repeated measures (.20) indicates a required sample size of 280 participants [45, 46]. We will increase this number by 10% (while ensuring that cell numbers are balanced) to reach the required sample size even if participants need to be excluded due to the criteria described below. The resulting sample would therefore comprise 308 participants.

As a prescreening criterion, we will require all participants to speak English as their native language and to be using headphones throughout the study. Participants who do not give consent will be screened out from the survey. Additionally, eligible participants will be asked to indicate whether they see any reason as to why their data should not be used for statistical analyses at the end of the study. If they ask for exclusion, we will not use their data for the analysis. We will analyze complete data sets only.

Monetary compensation will depend on the duration of the study, which will be pretested. We will pay a minimum of $7.25 per hour.

#### Design

Study 1 is set up as a mixed design with two between-subjects factors (secrecy: message framed as secret vs. not, and valence: information is positive vs. negative) and one within-subjects factor (audio recording number: one to five). Judgments of change in social distance between the participant and the person speaking in the audio recording, as well as between the participants and the person who had really told them secrets or news in the past will serve as dependent variables and will be measured with two different approaches (see Materials below).

#### Materials

All five stories in the audio recordings concern situations in which one person is sharing a piece of information about him- or herself with the participants (adjusted versions of the vignettes presented in S1 and S2 Files, see pretest), with this information being either framed as secret or not. This information is furthermore either positive (see S1 File) or negative (see S2 File). All vignettes have been designed to cover main categories of secrets identified in previous research [2] and are balanced in regards to names of the protagonists and the degree of control concerning the focus topic. Furthermore, the recordings will be obtained from five different speakers, who will each record one negative and one positive vignette, to keep voices constant across conditions.

All audio recordings will be presented in a randomized order. To assess our key dependent variables, it is important that we can differentiate between social distance per se and the variable of interest, namely perceptions of *change* in social distance between participants and the person in the audio recordings. To this end, we will first ask all participants “After having listened to the message, do you perceive that your relationship might have changed to some extent?” (answer options ranging from 1 = *not at all to* 7 = *very much*). Only if participants indicate a change (values greater than 1), we would then ask “After having listened to the message, do you perceive your relationship as closer or more distant than before?” (1 = *closer than before*; 7 = *more distant than before*). Furthermore, we will use the IOS scale [44], which serves as a pictorial measure of interpersonal closeness between two individuals, and will ask participants to evaluate the imagined social distance that would best describe the relationship.

To further minimize the risk of demand characteristics affecting our dependent variables, we will also ask participants to rate aspects of the exchange that are not related to social distance, such as the length of the conversation (1 = *very short*, 7 = *very long*), how soon the individuals would meet again (1 = *very soon*, 7 = *in a long while*), and whether the next encounter would take place in a more private or more public place (1 = *very public place*, 7 = *very private place*).

To assess our key dependent variables in regards to participants’ own past experiences, we will ask them to recall a situation in which a person shared either negative or positive secrets versus news with them (in line with one of the four conditions they had been assigned to). We will ask them to describe the situation without necessarily revealing the content of the information. Next, we will ask participants: “After that person told you this, did you perceive that your relationship might have changed to some extent?” (answer options ranging from 1 = *not at all to* 7 = *very much*). Only if participants indicate a change (values greater than 1), we would then ask “Did you perceive your relationship to be closer or more distant than before the person told you this?” (1 = *closer than before*; 7 = *more distant than before*).

#### Procedure

Participants will be welcomed to the study about “evaluation of behavior”. At the very beginning, we will explain that they will need to use headphones during the study. To ensure that they meet the study’s requirements, they will listen to a recording of a person describing a still life, and will then be asked to select all objects from a list of five that have been mentioned in the description. If participants correctly identify the objects mentioned, they can carry on with the study. After providing informed consent, they will then learn that the study aims to understand evaluations of other people’s behavior. To this end, they will be asked to imagine being on the phone with a friend and then listen to the audio recordings. In this call, the friend is describing a situation and shares some information with them. After listening to each recording, participants will be asked to rate how confidential the content of the exchange was (which will serve as a manipulation check) on a seven-point Likert scale (1 = *not confidential at all*; 7 = *very confidential*). Participants will first rate whether they perceive a change in the relationship, and if so, they will then rate the change in perceived social distance between themselves and the person, as well as the additional measures described above before listening to the second recording and so forth.

After having read and evaluated all recordings, participants will be asked to think about a past experience in which somebody shared a negative / positive piece of information / secret with them. Again, participants will rate whether they perceived a change in the relationship, and if so, rate the change in perceived social distance.

Last, participants will provide demographic information (gender and age) and will be asked whether they see any reasons as to why their data should not be included in the study. We will also ask them if they have an assumption what the underlying research question in the study might have been. At the end of the study, individuals will be debriefed and thanked for their participation.

### Data analytic plan and proposed analyses

After excluding all participants according to our a priori defined criteria (see above), we will use a repeated measures ANOVA to analyze whether our sample perceived recordings in the secret condition as more confidential than the recordings in the nonconfidential condition. This analysis will serve as a check to test whether our manipulation of secrecy was successful.

To test Hypotheses 1 and 2, we will calculate a repeated measures ANOVA with number of audio recordings as a repeated measure and secrecy as well as valence as between-subjects factors. Perceptions of change in social distance will serve as a continuous dependent variable. We will calculate this analysis once for the IOS scale measure and once for the direct evaluation of social distance in the relationship (for all recordings in which participants indicate that a change has occurred). To test our hypotheses with participants’ experiences, we will calculate an ANOVA with secrecy and valence as between-subjects factors. Perceptions of change in social distance will serve as continuous dependent variable. With both of these analyses, we will investigate whether participants perceive the social distance between themselves and another person as smaller when the exchanged information is secret compared to when it is not secret (main effect of secrecy). Furthermore, we will investigate whether this change differs depending on the valence of the content (interaction effect between secrecy and valence). If the interaction effect is significant, we will investigate the specific impact of valence crossed with secrecy with simple main effect analyses.

We will also analyze how many participants have correctly guessed our research question and whether excluding them affects our results.

## Study 2

The proposed Study 1 tests our idea that sharing secrets decreases social distance with a focus on the receiver. In Study 2, we aim at learning about changes in distance from an observer’s point of view and use a different methodological approach, namely vignettes. As is the case with recordings but not with participants’ experiences, written vignettes allow for describing a concrete situation while keeping both the content of the information exchange as well as previous levels of closeness constant. This provides us with a promising approach to study intimate relationships [47] and contributes to the internal validity of our study. To further explore the potential role of how identity-relevant the shared information is (and how this might impact ratings of change in closeness), we will ask participants to rate this aspect for each vignette at the end of the study.

Applying our hypotheses to this context, we expect that learning that two persons have exchanged a secret (rather than information only) results in lower perceived social distance between these individuals (Hypothesis 1). Furthermore, Study 2 will again investigate whether changes in social distance might depend on the nature of the information shared, meaning, whether it is positive or negative (Hypothesis 2).

### Methods

#### Participants

The study will be conducted as an online study advertised as a *study on the evaluation of behavior* via Prolific. Our a priori power analysis with an α-level of .05, a desired power of .95, and a small to medium effect size estimate (*f* = 0.15) while assuming a small correlation between repeated measures (.20) indicates a required sample size of 280 participants [45, 46]. We will increase this number by 10% (while ensuring that cell numbers are balanced) to reach the required sample size even if participants need to be excluded due to prescreening and the exclusion criteria described below. The resulting sample would therefore comprise 308 participants.

As a prescreening criterion, we will require all participants to speak English as their native language. Furthermore, participants who have previously worked on Study 1 will not be eligible to participate in Study 2. Participants who do not give consent will be screened out from the survey. Additionally, eligible participants will be asked to indicate whether they see any reason as to why their data should not be used for statistical analyses at the end of the study. If they ask for exclusion, we will not use their data for our analyses. We will analyze complete data sets only.

Monetary compensation will depend on the duration of the study, which will be pretested. We will pay a minimum of $7.25 per hour.

#### Design

Study 2 is set up as a mixed design with two between-subjects factors (secrecy: message framed as secret vs. not, and valence: information is positive vs. negative) and one within-subjects factor (vignette number: one to five). Perceived change in social distance between the interaction partners described in the vignettes will serve as dependent variable.

#### Materials

The complete set of vignettes with positive content can be found in S1 File and the set of vignettes with negative content can be found in S2 File. The instructions and questions within the study are similar to those of Study 1, see below.

#### Procedure

The procedure of Study 2 is identical to Study 1, except that participants will be asked to evaluate the anticipated change between the persons described in the vignettes (“After having read the interaction between [names of interaction partners], do you perceive that their relationship might have changed to some extent?”; answer options ranging from 1 = *not at all to* 7 = *very much*; and in case they indicated a change “Do you perceive the relationship between [names of interaction partners] as closer or more distant than before?”; 1 = *closer than before*; 7 = *more distant than before*).

In order to investigate potential differences between the content of the information shared (meaning the vignettes) in an exploratory fashion, we will further ask participants to reread the vignettes at the end of the study and rate how relevant they think the information is to the identity of the secret-sharer (1 = *very irrelevant*, 7 = *very relevant*).

### Data analytic plan and proposed analyses

Data analysis will be the same as in Study 1. After excluding all participants according to our a priori defined criteria (see above), we will use a repeated measures ANOVA to analyze whether participants perceived vignettes framed as secret compared to vignettes not framed as secret as more confidential. This analysis will serve as a check to test whether our manipulation of secrecy was successful.

With a repeated measures ANOVA, we will analyze whether participants perceive the change in social distance as different depending on whether the exchanges were framed as secrets or not (main effect of secrecy). Furthermore, we test whether the change in social distance depends on the valence of the content (interaction effect between secrecy and valence). If the interaction effect is significant, we will investigate the specific impact of valence crossed with secrecy with simple main effect analyses.

As exploratory analysis, we will further include the judgments on relevance of the information shared to the identity of the sharer as additional factor and analyze the data using a linear mixed-model.

We will also analyze how many participants have correctly guessed our research question and whether excluding them affects our results.

## Study 3

While Study 1 focuses on the perspective of the receiver and Study 2 on the perspective of an observer, Study 3 investigates the sharing of secrets from the sharer’s point of view in a lab setting where individuals are asked to either create social closeness or social distance by eventually sharing secrets. Study 3 thereby investigates whether participants’ choices and behavior regarding information sharing differ depending on the goal they have when interacting with others. We hypothesize that individuals are more likely to share secrets (vs. information) when they want to create social closeness compared to distance (Hypothesis 1), but that they choose to share more or fewer negative secrets in one of the two conditions (Hypothesis 2).

### Methods

#### Participants

The study will be conducted in the lab and advertised as *study on information sharing* via different university platforms. Data collection will take place within the COVID-19 situation, which might impact our study. Our a priori power analysis with an α-level of .05, a desired power of .80, and a medium effect size estimate for the within-between interaction term (*f* = 0.25) indicates a required sample size of 128 participants [45,46]. We will increase this number by 10% (while ensuring that cell numbers are balanced) to reach the required sample size even if participants need to be excluded due to the criteria described below. The resulting sample would therefore comprise 142 participants. We will collect data across a minimum time span of two months to ensure that we reach the sample size we aim for. If we can collect data from a larger sample during this time frame, we will do so to improve the statistical power of our tests. Data analysis, however, will only start after the completion of the data collection.

Participants who do not give consent will be screened out from the survey. Additionally, eligible participants will be asked to indicate whether they see any reason as to why their data should not be used for statistical analyses at the end of the study. If they ask for exclusion, we will not use their data for our analyses.

For an estimated participation time of 10 minutes, participants will receive chocolate or course credit as compensation.

#### Design

Study 3 is set up as a mixed design with one between-subjects factor (goal: closeness vs. distance) and one within-subjects factor (type of information: general information vs. positive secrets vs. negative secrets). Amount of shared information (zero to three) serves as dependent variable.

#### Materials and procedure

First, participants are asked to think about their own lives. They are asked to deliberate on general information that describes themselves, but also on secrets that they might have and would not feel comfortable to just share with anybody. They are next asked to narrow down these general thoughts to three pieces of general information about themselves, three positive secrets, and three negative secrets. Importantly, participants are *not* asked to write down these secrets, but to just think of them and provide a keyword, so they can later recognize them based on that keyword.

Second, participants learn that we would like to understand how they behave in a typical university context: a first day in class. They should imagine joining a new class in which they do not know anyone yet. We will then ask participants how they would act in such a situation when they would start working together with another student in the class. They are either told that they will continuously work with this person and therefore aim to create a feeling of closeness (closeness condition) or that they will probably not work with this student again throughout the course and therefore are not interested in creating a feeling of closeness (distance condition).

Third, we will ask them which information they would share with the other student. Participants will then see their nine keywords that they had previously entered and are asked to indicate which of the pieces of information they would share. Participants are asked to choose three pieces of information–a number that allows them to share only information, only positive secrets, only negative secrets, or any combination of those three types of information.

After collecting the data on sharing behavior, we will ask participants to complete a manipulation check. We will again show participants their keywords and ask how difficult they would find it to talk about this aspect with a stranger (manipulation check for secrecy) and how positive or negative this piece of information is (manipulation check for valence).

Finally, participants will be asked to provide demographic information (gender and age) and will be asked whether they see any reasons as to why their data should not be included in the study. At the end of the study, individuals will be debriefed and thanked for their participation.

### Data analytic plan and proposed analyses

After excluding all participants according to our a priori defined criteria (see above), we will conduct two repeated measures ANOVAs to analyze a) whether participants share more secrets in relation to general information in the closeness condition (interaction effect of goal and type of information, meaning general information versus secret), and b) whether the proportion of positive to negative secrets is different in the closeness than the distance condition (interaction effect of goal and valence of secret). If the interaction effects are significant, we will investigate the specific impact of type of information and valence of secrets crossed with goal with simple main effect analyses.

For the first analysis, we will collapse the positive and negative secrets and count the average number of shared secrets compared to general information shared to obtain our dependent variable. For the second analysis, we will compare the numbers of positive and negative secrets shared.

### Proposed timeline

Both authors are research assistants and are funded until at least December 2020. The first two proposed studies are web-based, allowing data collection to start directly after the completion of the review process of the Stage 1 submission. Data for Study 3 can be collected during the fall or spring term of the university. We anticipate that data collection will be completed within two to four months (if government and university policies allow lab-based data collection during the COVID-19 situation). Following the completion of data collection, we plan on finishing and documenting the data analyses and discussions within three months.

## Supporting information

S1 File Vignettes with positive information. (PDF)

S2 File Vignettes with negative information. (PDF)
