# Supplementary material for: Secretive and close? How sharing secrets may impact perceptions of distance
Source: PLoS One. 2020 Jun 11;15(6):e0233953. doi: 10.1371/journal.pone.0233953 (PMC7289348; doi:10.1371/journal.pone.0233953)
Supplement: S1 File — (PDF) [file pone.0233953.s001.pdf]

Supporting Information

S1 File. Vignettes with positive information.

Vignette 1

Helen tells her colleague Leonie that she has decided to accept an incredible job offer in New York City. In general, she is very happy with her current job, but one of the most renowned fashion agencies took notice of her last year and she could not believe they would offer her a job right away. In addition, New York City has always been a city she has dreamed of. Helen will therefore soon quit her current job and is very excited when she tells Leonie about it.

Secret condition add-on: Yet, she asks Leonie not to tell anyone.

Vignette 2

Anna tells Laura that her boyfriend has proposed to her. She had been waiting for this for such a long time. After all, they have been together for more than five years and to Anna it seemed clear from the beginning that she wanted to spend the rest of her life with this man. She thinks that her boyfriend might just have been too busy with his job so that he did not even have the time to think about marrying. Now Anna is even happier that he has finally asked her. “I couldn’t wait to share the news with you!”, she tells Laura happily.

Secret condition add-on: Anna, however, asks Laura not to tell anyone about it.

Vignette 3

Tom tells David about an incredible occurrence. Throughout his childhood, he believed his father had abandoned him and did not want any contact whatsoever. But yesterday, a lawyer came to inform him that his father mentioned him in his will and left him a large sum of money! “Just now

that I'm on such a tight budget!", says Tom and can hardly believe his luck. Suddenly, he sees his future from a completely new angle and is incredibly excited, as so much seems possible for him now.

Secret condition add-on: However, Tom asks David to treat it confidentially and to not tell anyone how he happened to have so much money.

Vignette 4

Tina is so excited when she tells Melanie that her greatest childhood dream is just within her reach. She signed up to audition at a very renowned ballet school and they have just told her that she has made it to the final round. She never believed she could outshine so many fellow applicants! "Now I need to practice even harder for the final in two weeks. Fifteen dancers are nominated, and the jury will only accept five of us!" Tina is thus very nervous about the event.

Secret condition add-on: She asks Melanie to promise not to tell anyone though.

Vignette 5

Joshua tells Céline he could not believe his luck. He and his wife had been hoping to have a child for years now. They went through so many medical assessments together that they stopped counting and they also tried alternative therapies, but she would just not get pregnant. Their hope that they could have a baby diminished with every day until they finally gave up trying. This was around a year ago. "And now guess what we found out yesterday!", Joshua says, his eyes sparkling. Apparently, after they stopped trying and thinking about it all the time, it worked and the pregnancy test was positive.

Secret condition add-on: However, he emphasizes that Céline should not tell anyone about his wife's pregnancy yet.
