# Supplementary material for: Secretive and close? How sharing secrets may impact perceptions of distance
Source: PLoS One. 2020 Jun 11;15(6):e0233953. doi: 10.1371/journal.pone.0233953 (PMC7289348; doi:10.1371/journal.pone.0233953)
Supplement: S2 File — (PDF) [file pone.0233953.s002.pdf]

Supporting Information

S2 File. Vignettes with negative information.

Vignette 1

Joshua confides to Céline that he is not doing well at all. Actually, it started several months ago that he was so tired at times that he hardly managed to get out of bed in the morning. Back then, things at the office became overwhelming he was afraid to lose his job. “I did not really think about it that much”, he says, and tells her that he got himself some cocaine to overcome that low phase. Before he could even realize, the addiction got hold of him, and now he is afraid that his whole life is getting out of hand.

Secret condition add-on: Joshua urges Céline to promise that she keeps this secret to herself.

Vignette 2

Helen tells her colleague Leonie that she made a terrible mistake. Out of jealousy, she intentionally started bad rumors about her friend Louise. Louise is now devastated and some say she the bad rumors are the reason she lost her job. It was over such a small matter, and now everything has gotten out of hand, Helen says with a downcast voice. She also fears that Louise might take revenge if she found out what Helen did.

Secret condition add-on: Leonie therefore should not tell anyone that Helen was the one who started the bad rumors.

Vignette 3

Tom talks to David about his work. Tom really used to like his job as a product manager. Half a year ago, however, his superior quit and was replaced by one of his colleagues. In the beginning,

everything seemed to run smoothly, but over time his new superior became very dominant and started to put a lot of pressure on the whole team. “It is nothing like it used to be, I can hardly bear his presence!”, Tom says. He is now even considering to quit and to look for a new job.

Secret condition add-on: David really should not tell anyone about these thoughts, Tom insists.

Vignette 4

Anna tells Laura about her marriage. She had been feeling very distant towards her husband and was not sure whether this was just a phase or whether it was a real change in their relationship. When she could not bear it any longer, she asked him whether something was wrong. “You know, it is not the first time this happened”, Anna says and explains that her husband has been cheating on her again. For years they have been trying to work on their relationship and Anna always thought she wanted to give him another chance. But now that he betrayed her for the third time, she is seriously thinking of getting divorced and has already contacted a lawyer.

Secret condition add-on: Anna makes Laura promise to keep the secret to herself.

Vignette 5

Tina tells Melanie about her success at work. For a few months now, her company has reported great profit and has become known nationwide. This was due to a fantastic publicity campaign that she initiated. “Well, in fact it was an employee’s idea, but it just looks so much better if I claim it to be my own idea.”

Secret condition add-on: Yet, Melanie needs to promise not to tell anyone about the swindle.
